# Association of Hospital Interoperable Data Sharing With Alternative Payment Model Participation

**DOI:** 10.1001/jamahealthforum.2021.5199

**Published:** 2022-02-18

**Authors:** A Jay Holmgren, Jordan Everson, Julia Adler-Milstein

**Affiliations:** 1 University of California, San Francisco; 2 Vanderbilt University Medical Center, Nashville, Tennessee

## Abstract

**Importance:**

Interoperable patient data exchange across hospitals remains an important policy goal for reducing costs and improving the quality of care. Congress designated 2018 as the goal for nationwide interoperability, and policy makers hoped that aligning financial incentives via alternative payment models (APMs) would help achieve that goal.

**Objective:**

To measure interoperability progress since 2014, assess the association between alternative payment model participation and hospital engagement in interoperable data sharing from 2014 to 2018, and evaluate hospital-reported barriers to interoperability in 2018.

**Design, Setting, and Participants:**

This cohort study included nonfederal acute care hospitals in the US from January 2014 to December 2018 that responded to the American Hospital Association Annual Survey. Data were analyzed from October 2019 through March 2021.

**Exposures:**

Participation in an APM, including accountable care organizations, bundled payments, or patient-centered medical homes.

**Main Outcomes and Measures:**

Hospital engagement in all 4 domains of interoperability: finding/querying for data, sending data electronically, receiving data electronically, and integrating electronic patient data from external care delivery organizations.

**Results:**

The sample included 3928 hospitals in the US from January 2014 to December 2018. Progress across interoperability domains was uneven, 2430 (88.3%) hospitals sending and 2115 (76.9%) receiving patient data electronically in 2018. However, only 1249 (45.4%) hospitals engaged in all 4 domains of interoperability in 2018, and growth between 2014 and 2018 was slow. There was no evidence that participation in APMs was associated with interoperability, with multivariate models suggesting that participation in an APM was associated with only a non–statistically significant 1–percentage point increase in interoperability engagement (β = 0.01; 95% CI, −0.01 to 0.03). The most commonly cited barrier to interoperability was challenges associated with sharing data across different electronic health record vendors.

**Conclusions and Relevance:**

In this cohort study of hospital interoperability, fewer than half of US hospitals were engaged in interoperable data exchange in 2018. There was no observable evidence that hospital APM participation was associated with interoperability engagement. Many hospitals report technical and governance challenges to data sharing that are unlikely to be addressed by the alignment of financial incentives alone.

## Key Points

**Question:**

How much progress did US hospitals make toward building a nationally interoperable health system from 2014 to 2018, and was aligning financial incentives via alternative payment models associated with interoperability?

**Findings:**

In this cohort study of 3928 US hospitals, progress in interoperability was slow, with fewer than half of hospitals reporting that they engaged in all 4 domains of interoperability by 2018. No evidence of an association between alternative payment model participation and interoperable data sharing was found.

**Meaning:**

The results of this study suggest that building a nationally interoperable health care system remains a complex and challenging task that requires more than just alignment of financial incentives via voluntary payment reform programs.

## Teaser

This cohort study examines interoperability progress, the association between alternative payment model participation and hospital engagement in interoperable data sharing, and hospital-reported barriers to interoperability.

## Introduction

Following the passage of the US Health Information Technology for Economic and Clinical Health (HITECH) Act of 2009, substantial public and private effort has focused on increasing interoperable data exchange to provide clinicians access to relevant patient records at the point of care, regardless of where the records were originally created.^1,2,3^ The potential benefits of nationwide interoperability are substantial, including improved patient outcomes and reductions in unnecessary and duplicative care.^4,5,6^ However, early progress after HITECH was slow, and as of 2014, fewer than one-quarter of US hospitals were engaged in all 4 of the interoperability domains identified by the Office of the National Coordinator for Health Information Technology (ONC): electronically finding (querying), sending, receiving, and integrating information into electronic health records (EHRs).^7,8,9^ To spur greater interoperability progress, Congress in 2015 declared a “national objective to achieve widespread exchange of health information through interoperable certified EHR technology nationwide by December 31, 2018.”^10^

One policy mechanism by which Congress intended to achieve the goal of national interoperability by 2018 was via programs designed to better align financial incentives to encourage data sharing known as alternative payment models (APMs), as described in the US Medicare Access and Children's Health Insurance Program Reauthorization Act (MACRA) of 2015. While other efforts to encourage interoperability were also underway during this period, policy makers and researchers focused on these payment reform initiatives as some of the most promising potential drivers of interoperability.^11^ In contrast, health information technology (IT) provisions of the 21st Century Cures Act took a targeted approach by directly advancing technology, standards, and governance models with the aim of reducing the technical and administrative complexity of interoperability and addressing specific barriers, such as information blocking.

Assessing progress against the 2018 congressional objective could reveal the trajectory of interoperability and appropriateness of targeting lagging areas. Stronger incentives for data sharing were expected to arise from the spread of risk-bearing population-based and episode-based APMs,^12,13^ such as accountable care organizations (ACOs), patient-centered medical homes (PCMH), and bundled payments.^14^ While these models were designed to improve quality and reduce costs, they were expected to promote interoperability to achieve those goals.^7,15^

We sought to bring new evidence to assess progress in interoperability using the HITECH/MACRA policy framework. We used national hospital data to track interoperability progress from 2014 to 2018 and compare this progress between hospitals that participated in APMs and those that did not. We also assessed hospital-reported barriers to interoperability and compared differences in barriers between hospitals that did and did not participate in APMs. These analyses provide insights regarding the challenges to interoperability that need to be addressed in ongoing 21st Century Cures policy making.^16^

## Methods

### Data and Sample

We used data from 2014 to 2018 from the American Hospital Association (AHA) Annual Survey and IT Supplement to capture hospitals’ engagement in interoperability, participation in APMs, and other hospital characteristics.^17^ The surveys are sent annually to every hospital in the US with a request that the chief executive officer (or chief information officer for the IT supplement) complete the survey or delegate it to the most knowledgeable person. The 2018 IT Supplement survey collected data from January to May 2019 regarding hospital IT status at the end of 2018. The study used secondary data on organizations and was thus exempt from human participants research approvals. This study followed the Strengthening the Reporting of Observational Studies in Epidemiology (STROBE) reporting guidelines for all estimates reported.

To measure trends over time, we created a panel of hospitals that responded to the AHA IT Supplement at least once from 2014 to 2018. We limited the sample to nonfederal acute care hospitals, as those were subject to HITECH Act and considered eligible hospitals for the Meaningful Use and Promoting Interoperability programs. We matched these data to AHA Annual Survey data from 2014 to 2018 to capture characteristics, including the number of beds, ownership, teaching status, location, system membership, and participation in 3 payment reform programs. The sample comprised 3928 unique hospitals during a period of 5 years and a total of 13 883 hospital-year observations.

### Measures

#### Interoperability Domains

We created 4 dichotomous measures to capture hospital engagement in each of the 4 domains of interoperability as defined by ONC. These functionality-based, technology-agnostic domains capture the core elements of interoperable data exchange required for clinical use in which care delivery organizations need to share information electronically,^9^ and the AHA IT Supplement began measuring these in 2014.

Sending and receiving information facilitates electronic data exchange for planned transitions of care, including discharges to an outpatient setting or referrals to another organization. Finding (querying) data, the ability to search or query for patient data from outside organizations, is a necessary capability for unplanned care transitions, such as emergency care.^18,19^ Integrating patient information is a critical capability that distinguishes interoperability from health information exchange; data from outside organizations can be integrated or incorporated into the EHR without manual effort.^2,20^ We defined these variables as consistent with previous studies and ONC standards.^7,21^ Full variable descriptions are available in eMethods 1 in the Supplement.

#### APM Participation

We created dichotomous measures of our treatment variable, participation in each of the 3 APMs, using questions from the AHA Annual Survey. The 3 alternative payment models we included were ACOs, PCMH, and bundled payments involving inpatient, physician, or post–acute care bundles. The ACO and PCMH data were available during each year from 2014 to 2018, while bundled payment data were available for 2015 to 2018. Hospitals that participated in 1 or more APMs in a year were considered an APM participant. Full variable descriptions are available in eMethods 2 in the Supplement.

#### Barriers to Interoperability

We measured barriers to interoperability in 2018 using the question “Which of the following issues has your hospital experienced when trying to electronically (not eFax) send, receive, or find (query) patient health information to/from other care settings or organizations?” with 12 possible barriers to select yes or no options. Hospitals that responded yes to any of the subquestions were considered as experiencing that barrier. We limited the sample to hospitals that reported engaging in all 4 domains of interoperability to minimize availability bias and conducted robustness tests with all hospitals, as well as hospitals that reported at least sending and receiving data.

#### Hospital Characteristics

We included several hospital characteristics based on previous studies of interoperability.^7,22^ These included level of EHR adoption (less than basic, basic EHR, or comprehensive EHR),^23^ membership in a regional health information exchange organization,^24^ size (by number of beds), teaching status, membership in a health care system, urban or rural location, and geographic region. We report descriptive statistics of these, as well as hospital EHR vendors, in eTable 1 in the Supplement.

### Statistical Analysis

We calculated the proportion of hospitals that engaged in each of the 4 domains of interoperability, as well as all 4 domains, in each year from 2014 to 2018. We assessed progress in all 4 domains by hospital characteristics, comparing the first and last years (2014 and 2018). All measures used weights generated by an inverse probability model to account for nonresponse bias and create nationally representative estimates.^25^

To compare APM and non-APM hospitals, we calculated engagement in interoperability during each year by APM participation status, with χ^2^ tests for statistical significance. We created 2 ordinary least squares linear probability models, with the dependent variable as the dichotomous measure of whether a hospital engaged in all 4 domains of interoperability and the independent variable of interest as participation in any APM in the first model and each of the 3 APMs individually in the second model. Both models included hospital fixed effects to control for time-invariant omitted variables and year fixed effects to control for secular trends. Both models included controls for time-varying hospital characteristics, including EHR adoption level, health information organization participation, and system membership, as well as robust standard errors clustered at the hospital level. We conducted robustness tests and alternative specifications, including difference-in-differences models that used the Callaway and Sant’Anna estimator designed for varying treatment timing^26^ (eMethods 3 in the Supplement).

Finally, we compared the proportion of hospitals reporting each barrier to interoperability in 2018 for APM and non-APM hospitals using χ^2^ tests. All statistical tests used a 2-sided α of .05 for statistical significance. Analysis was performed from October 2019 through March 2021 using Stata, version 17 (StataCorp).

## Results

### Interoperability Progress

**Figure 1.**
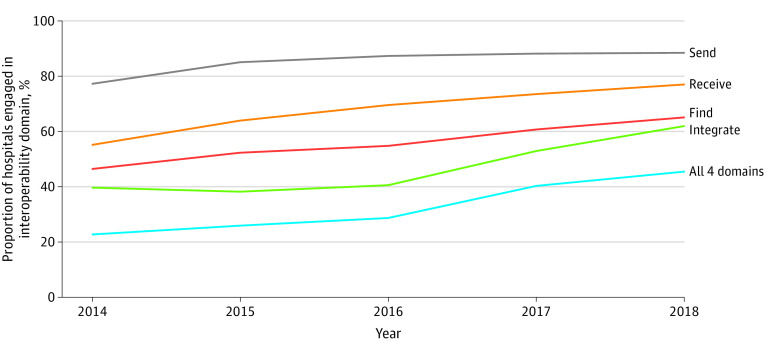
Proportion of US Hospitals Engaging in Interoperability by Domain Analysis of American Hospital Association Annual Survey and IT Supplement data, 2014 to 2018.

The AHA Annual Survey IT Supplement response rate for nonfederal acute care hospitals was 64% (n = 3540) for 2018. As of 2018, 1249 US hospitals (45.4%) engaged in all 4 domains of interoperability. The most widely adopted domain in 2018 was sending data (2430 hospitals [88.3%]), followed by receiving data (2115 [76.9%]), and finding data (1785 [64.9%]). Integrating data without manual intervention was the least commonly adopted domain, with 1702 hospitals (61.8%) reporting they had the capability to do so in 2018 (Figure 1).

The number of hospitals that reported that they engaged in all 4 domains of interoperability increased by 22.8 percentage points from 2014 when only 635 hospitals (22.6%) reported they were had all 4 capabilities. The most progress from 2014 to 2018 was in the integrate domain, with a 22.3 percentage point increase from 39.5% (n = 1110) in 2014. Receiving data had the next most progress with 21.9 percentage points, from 54.9% (n = 1544) in 2014, followed by finding data, with an 18.6 percentage point increase from 46.2% (n = 1299) in 2014. Sending data increased 11.2 percentage points, from 77.1% (n = 2166) of hospitals in 2014.

**Figure 2.**
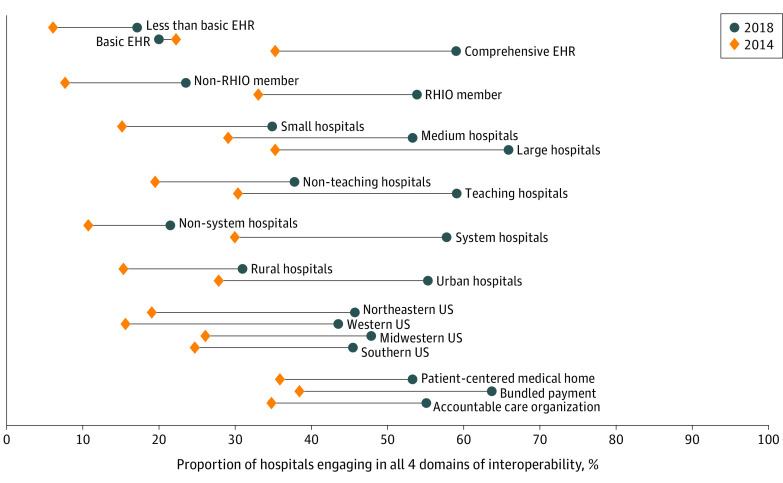
Interoperability Progress From 2014 to 2018 by Hospital Characteristics Analysis of American Hospital Association Annual Survey and IT Supplement data, 2014 to 2018. Barbells represent change in hospital engagement in all 4 domains of interoperability from 2014 to 2018 as stratified by hospital demographic characteristics. Orange diamonds indicate interoperability engagement among hospitals in that category in 2014, while dark blue circles represent the same in 2018. EHR indicates electronic health record; RHIO, regional health information exchange organization.

Comparing engagement in all 4 domains of interoperability from 2014 to 2018 by hospital characteristics, large hospitals made the most progress, with a 30.6 percentage point increase from 2014 to 2018 (*P* < .001). Teaching hospitals (28.7 percentage point increase), system hospitals (27.8 percentage points), and hospitals located in urban areas (27.5 percentage point increase) also made substantial progress. Hospitals with a basic EHR in the measure of EHR sophistication saw a 2.2 percentage point decrease in interoperability engagement (Figure 2).

### Interoperability by APM Participation

**Figure 3.**
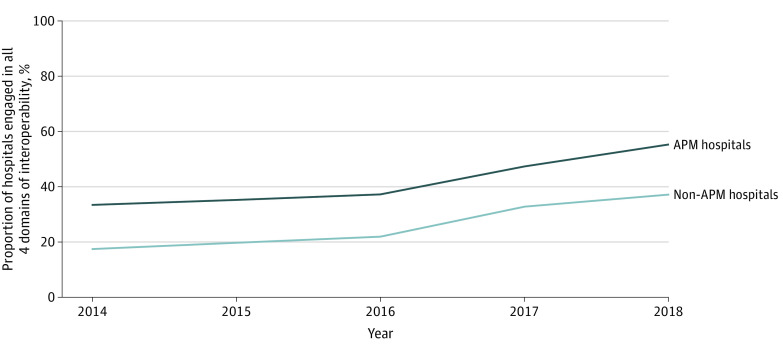
Interoperability Progress From 2014 to 2018 by Alternative Payment Model (APM) Participation Analysis of American Hospital Association Annual Survey and IT Supplement data, 2014 to 2018.

In 2014, APM hospitals engaged in interoperability at a significantly higher level than non-APM hospitals (33.5% [n = 281] for APM hospitals and 17.6% [n = 320] for non-APM hospitals; *P* < .001). The APM hospitals saw a 21.9 percentage point increase up to 55.4% (n = 691) in 2018 (an average increase of 5.5 percentage points per year), while non-APM hospitals increased by 19.7 percentage points over time (an average increase of 4.9 percentage points per year) to reach 37.2% (n = 573) in 2018 (Figure 3).

In multivariate models with hospital and year fixed effects, we found no evidence that participation in an APM was associated with interoperability engagement, regardless of the independent variable as participation in any APM (β = 0.01; 95% CI, −0.01 to 0.03) or separately for PCMH (β = 0.01; 95% CI, −0.02 to 0.03), bundled payments (β = −0.01; 95% C, −0.04 to 0.02), and ACOs (β = 0.01; 95% CI, −0.01 to 0.04) (Table 1). Full model results, robustness checks with alternative estimators, and model designs and diagnostic tests are available in the eMethods 3 in the Supplement which found similar results.

**Table 1.** Association Between APM Participation and Hospital Engagement in Interoperability

Dependent variable: hospital engagement in all 4 domains of interoperability	Coefficient (95% CI)^a^	*P* value
Model 1: any APM		
APM participation	0.01 (−0.01 to 0.03)	.30
Basic EHR	0.01 (−0.01 to 0.03)	.30
Comprehensive EHR	0.08 (0.06 to 0.11)	<.001
RHIO participation	0.12 (0.10 to 0.15)	<.001
Member of a health system	<0.01 (−0.05 to 0.05)	.92
Model 2: specific APMs		
Patient-centered medical home	0.01 (−0.02 to 0.03)	.69
Bundled payment program	−0.01 (−0.04 to 0.02)	.45
Accountable care organization	0.01 (−0.01 to 0.04)	.30
Basic EHR	0.01 (−0.01 to 0.03)	.30
Comprehensive EHR	0.08 (0.06 to 0.11)	<.001
RHIO participation	0.12 (0.10 to 0.15)	<.001
Member of a health system	<0.01 (−0.05 to 0.05)	.94

Abbreviations: AHA, American Hospital Association; APM, alternative payment models; EHR, electronic health record; RHIO, regional health information exchange organization.

^a^ Analysis of AHA Annual Survey and IT Supplement data, 2014 to 2018. Models 1 and 2 are ordinary least squares linear probability models and include hospital and year fixed effects and robust standard errors clustered at the hospital level. Coefficients can be interpreted as percentage point changes in likelihood of interoperability.

### Barriers to Interoperability

Several barriers were reported by at least half of hospitals, including experiencing greater challenges exchanging data across different vendor platforms (735 [80.8%]); providers (the survey instrument uses the term *provider*, which can be ambiguous as to whether the respondents were indicating an individual clinician or practice; a care delivery organization, such as a hospital or clinic; or both) with which the hospital shares patients not typically exchanging patient data with them (771 [72.5%]); providers with which the hospital would like to send patient information electronically having an EHR, but it lacks the technical capability to receive information (834 [66.4%]); and difficulty locating the address of the provider to send information (697 [55.4%]) (Table 2). We found several differences in the prevalence of reported barriers between APM and non-APM hospitals (Table 2). Hospitals participating in APMs were more likely to report difficulty locating a provider’s address to send the information (65.1% [n = 442] compared with 44.1% [n = 255] for non-APM; *P* < .001), that providers that they wanted to send data to did not have an EHR (55.5% [n = 376] vs 39.6% [n = 229] non-APM; *P* < .001), difficulty matching or identifying the correct patient between systems (54.0% [n = 308] vs 37.0% [n = 183] non-APM, *P* < .001); and many recipients of their electronic care summaries reporting that the information was not useful (43.8% [n = 297] vs 32.9% [n = 190] non-APM; *P* < .001). The APM hospitals were less likely to report that providers with which they share patients did not exchange data with them (66.3% [n = 378] compared with 79.6% [n = 393] for non-APM; *P* < .001) and that they had to develop customized interfaces to electronically exchange health information (34.4% [n = 201] vs 43.5% [n = 141] non-APM; *P* = .01). We also found that, controlling for observable hospital characteristics, APM hospitals reported more barriers than non-APM hospitals (eTable 2 in the Supplement). We found similar results when expanding to include all hospitals (eTable 3 in the Supplement) and hospitals that engaged in at least sending and receiving data (eTable 4 in the Supplement).

**Table 2.** Barriers to Interoperability Among APM and Non-APM Hospitals Among Hospitals Engaged in All 4 Domains of Interoperability in 2018

Characteristic	No. (%)^a^			*P* value
	All hospitals	Non-APM hospitals	APM hospitals	
APM hospitals more likely to report barrier				
Difficult to locate the address of the provider^b^ to send the information (eg, lack of provider directory)	697 (55.4)	255 (44.1)	442 (65.1)	<.001
Providers we would like to electronically send patient health information to do not have an EHR or other electronic system with capability to receive the information	605 (48.2)	229 (39.6)	376 (55.5)	<.001
Difficult to match or identify the correct patient between systems	491 (46.1)	183 (37.0)	308 (54.0)	<.001
Many recipients of our electronic care summaries (eg, CCDA) report that the information is not useful	487 (41.5)	190 (32.9)	297 (43.8)	<.001
APM hospitals less likely to report barrier				
There are providers with which we share patients with that do not typically exchange patient data with us	771 (72.5)	393 (79.6)	378 (66.3)	<.001
We had to develop customized interfaces to electronically exchange health information	342 (37.6)	141 (43.5)	201 (34.4)	.01
APM and non-APM hospitals equally likely to report barrier				
Experience greater challenges exchanging (eg, sending/receiving data) across different vendor platforms	735 (80.8)	261 (80.7)	474 (80.9)	.58
Providers we would like to electronically send patient health information to have an EHR; however, they lack the technical capability to receive the information	834 (66.4)	390 (67.5)	444 (65.5)	.99
We have to pay additional costs to send/receive data with care settings/organizations outside our system	376 (38.7)	132 (40.8)	244 (41.8)	.82
Cumbersome workflow to send (not eFax) the information from our EHR system	210 (16.7)	95 (16.4)	115 (17.0)	.62
No technical capability to electronically receive from outside providers	66 (6.2)	31 (6.2)	35 (6.1)	.94
No technical capability to electronically send to outside providers	32 (2.6)	18 (3.2)	14 (2.1)	.28

Abbreviations: AHA, American Hospital Association; APM, alternative payment model; CCDA, consolidated clinical document architecture; EHR, electronic health record.

^a^ Analysis of AHA Annual Survey and IT Supplement data, 2014 to 2018. Denominators can vary across questions, as nonrespondents were excluded and not all hospitals responded to every question.

^b^ The survey instrument uses the term *provider*, which can be ambiguous as to whether the respondents were indicating an individual clinician or practice; a care delivery organization, such as a hospital or clinic; or both. Future data collection efforts should make an effort to clarify this distinction.

## Discussion

We measured interoperability progress from 2014 to 2018 and found that engagement was limited, with fewer than half of hospitals engaging in all 4 domains by 2018. Progress was slow, with engagement increasing at an average rate of 5.7 percentage points per year. Compared with EHR adoption, which rose quickly during the decade following the passage of the HITECH Act in 2009, at the current rate, it will take until 2027 to achieve nationwide engagement in all 4 domains of interoperability, which is 18 years after the passage of HITECH. The policy efforts in the HITECH/MACRA era, from technical requirements in EHR certification to payment incentives in APMs, did not deliver on the goal of national interoperability by 2018. Achieving widespread interoperability has proven to be a much more complex challenge compared with EHR adoption and requires a more comprehensive approach.

The results of this analysis of APMS suggest that they were not associated with more substantial increases in interoperability among participating hospitals. While APM hospitals had a higher level of interoperability compared with non-APM hospitals in 2014, engagement in all 4 domains of interoperability grew at a similar rate in APM and non-APM hospitals during the 5-year study period. Similarly, the 2-way fixed-effects regression model that controlled for time-invariant unobserved hospital characteristics found no association between participating in an APM and engagement in all 4 domains of interoperability.

There are several reasons why APM participation may not have been associated with interoperability engagement. It may be that the incentives are too weak, with an insufficient proportion of hospital revenue connected to these programs to justify new investment in interoperability. Given that most APM payment is based on fee-for-service programs, with only upside rewards rather than more aggressive downside risk arrangements, APM incentives may not be strong enough to change hospital behavior substantially.^27^ It may also be that the incentives are too diffuse; even if APM participation affects hospital decision-making, hospital leaders may choose to focus on reducing costs in other ways, such as reducing utilization or shifting referrals to lower-cost care facilities.^28^ Evidence on ACOs suggests that care coordination is unlikely to be significantly associated with savings, and ACO participants target areas of unnecessary care that are more within their control.^29,30^

Such challenges are underscored by our results associated with barriers to interoperability. The most common barriers were technical and governance issues, such as cross-vendor data sharing, exchange partners lacking the technical capacity to receive data, and clinician and patient matching. The differences between APM and non-APM hospitals highlight the specific limitations of the value-based payment approach: while APM hospitals were less likely to report exchange partners being unwilling to share data or charging for custom interfaces to do so, they were equally or more likely to report several of the technical and governance barriers, such as difficulties exchanging across different EHR vendors, and APM hospitals may encounter these barriers more often, as they have incentives to fill in information gaps for care coordination. This suggests that value-based payment models aligned financial incentives for sharing data, but technical barriers hampered interoperability progress.

Rulemaking following the 21st Century Cures Act^31^ provides a framework for the next generation of interoperability that may address some of these barriers. This includes setting standards for application programming interface (API) implementation by EHR vendors, which can reduce the technical complexity of exchange between clinicians as API-based queries can be standardized across exchange partners. Advancing certified EHRs to include standard-based APIs may address other commonly cited barriers, such as difficulties with cross-vendor exchange.^32^ Another effort to simplify the exchange outlined in the Cures Act is the Trusted Exchange Framework and Common Agreement. It seeks to enable exchange of patient health information across health information networks, reducing the need to participate in multiple networks to exchange with trading partners that may participate in different networks. The regulations also prohibit information blocking by EHR vendors and care delivery organizations, which is the practice of preventing interoperability for reasons such as not sharing data with market competitors, which may address the issue of care delivery organizations choosing to not share patient data, a barrier cited by more than 70% of hospitals in this study. This prescriptive approach also contrasts with the HITECH Act, which focused on primarily on EHR adoption rather than requiring data exchange across those EHRs, and it is critical to measure interoperability progress and evaluate the effectiveness of these new policies that more directly target the technological barriers and mandate sharing of structured data elements.^33^

### Limitations

Our work should be interpreted with several limitations in mind. First, we used self-reported survey data and were unable to verify the accuracy of responses, although the AHA Annual Survey and IT Supplement are widely used and have been previously validated against external sources.^34^ Second, there are multiple ways to measure interoperability, such as participation, breadth, and volume. We focused on 4 federally prioritized domains of interoperability, but were unable to observe variability in implementation and use. Future research should examine differences across exchange methods. For example, the process of integrating data into the EHR without manual intervention may be different between hospitals using different methods of data sharing. Similarly, there are dimensions of APM participation that our data were unable to capture, such as whether a hospital participated in a Medicare or commercial ACO and the specific bundled payment services. Third, while we use a 2-way fixed-effects model to control for time-invariant omitted variable bias, time-varying confounders may have biased our results. We tested for these to the extent possible using measures described in eMethods 3 in the Supplement. Therefore, we were unable to fully establish either the size or directionality of a causal effect. Fourth, it is possible that our 2-way fixed-effects model is not appropriately powered to detect an association of APM participation with interoperability, resulting in a bias toward the null result. However, given that we found significant associations between other time-varying hospital characteristics, if APMs had an effect not detected in our model, it would be so small that it would not serve as an effective policy mechanism to incentivize interoperability. Finally, our specific analysis focuses on evaluating the association of APM participation with interoperability, and while the results inform speculation regarding other factors that may be associated with interoperability, the study design did not allow an in-depth analysis. Previous work has explored how these characteristics are associated with interoperability,^7,21,35,36,37^ and future research should continue to study how EHR vendor, size, teaching status, or location affect hospital engagement in interoperability.

## Conclusions

In this cohort study of hospital interoperability, we found fewer than half of hospitals were engaged in all 4 domains of interoperability in 2018. Interoperability progress was slow; if progress continued at the same rate, it would take until 2027 to achieve nationwide interoperability. We found no evidence that APM participation was associated with interoperable data sharing. The new framework to encourage data sharing through the 21st Century Cures Act may address remaining barriers to interoperability, such as technical challenges and issues with data governance.

## Notes

Supplement. **eMethods 1.** Full AHA Survey Questions for the Four Domains of Interoperability **eMethods 2. **Full AHA Survey Questions for Alternative Payment Model Participation **eTable 1. **Descriptive Statistics for Hospital Demographics and EHR Vendors **eMethods 3. **Technical Appendix and Robustness Tests for Two-Way Fixed Effects Design **eTable 2. **Association of APM Participation and Number of Hospital-Reported Barriers to Interoperability **eTable 3. **Hospital Reported Barriers to Interoperability on Full Hospital Sample (2018) **eTable 4. **Hospital Reported Barriers to Interoperability, Limited to Hospitals Who Engaged in the Send and Receive Domain (2018)
