# Supplementary material for: Association of Hospital Interoperable Data Sharing With Alternative Payment Model Participation
Source: JAMA Health Forum. 2022 Feb 18;3(2):e215199. doi: 10.1001/jamahealthforum.2021.5199 (PMC8903122; doi:10.1001/jamahealthforum.2021.5199)
Supplement: Supplement. — eMethods 1. Full AHA Survey Questions for the Four Domains of Interoperability eMethods 2. Full AHA Survey Questions for Alternative Payment Model Participation eTable 1. Descriptive Statistics for Hospital Demographics and EHR Vendors eMethods 3. Technical Appendix and Robustness Tests for Two-Way Fixed Effects Design eTable 2. Association of APM Participation and Number of Hospital-Reported Barriers to Interoperability eTable 3. Hospital Reported Barriers to Interoperability on Full Hospital Sample (2018) eTable 4. Hospital Reported Barriers to Interoperability, Limited to Hospitals Who Engaged in the Send and Receive Domain (2018) [file jamahealthforum-e215199-s001.pdf]

Supplemental Online Content

Holmgren AJ, Everson J, Adler-Milstein J. Association of hospital interoperable data sharing with alternative payment model participation. *JAMA Health Forum*. 2022;3(2):e215199. doi:10.1001/jamahealthforum.2021.5199

eMethods 1. Full AHA Survey Questions for the Four Domains of Interoperability
eMethods 2. Full AHA Survey Questions for Alternative Payment Model Participation
eTable 1. Descriptive Statistics for Hospital Demographics and EHR Vendors
eMethods 3. Technical Appendix and Robustness Tests for Two-Way Fixed Effects Design
eTable 2. Association of APM Participation and Number of Hospital-Reported Barriers to Interoperability
eTable 3. Hospital Reported Barriers to Interoperability on Full Hospital Sample (2018)
eTable 4. Hospital Reported Barriers to Interoperability, Limited to Hospitals Who Engaged in the Send and Receive Domain (2018)

This supplemental material has been provided by the authors to give readers additional information about their work.

© 2022 Holmgren AJ et al. *JAMA Health Forum.*

eMethods 1. Full AHA Survey Questions for the Four Domains of Interoperability

Finding (Querying): We identified hospitals that queried for data from external sources through the question "Do providers at your hospital query electronically for patients' health information (e.g., medications, outside encounters) from sources outside your organization or hospital system?" Hospitals that responded "yes" to this question were considered to be engaged in finding data.

Sending and Receiving: We identified hospitals that sent and received data through the question, "When a patient transitions to another care setting or organization outside your hospital system, how does your hospital routinely send and/or receive a summary of care record?" Respondents could choose from several options: "secure messaging using EHR (via direct or other secure protocol)," "provider portal," or "via health information exchange organization or other third party." Hospitals that responded "yes" to one or more options were considered to be electronically sending or receiving data. In 2017, the question was updated to "When a patient transitions to another care setting organization outside your hospital system, how often does your hospital use the following methods to send and/or receive a summary of care record?" and response options were updated to "Provider portal for access to EHR system", "Interface connection between EHR systems (e.g. HL7 interface)", "Direct access to EHRs (via remote or terminal access)", "Standalone HISP or HISP provided by a third party that enables secure messaging (such as DIRECT)", "Community (regional, state, or local) health information exchange organization", "Single EHR vendor network (use your EHR vendor's name that enables connection to vendor's other users such as Epic's Care Everywhere)", "Multi-EHR vendor networks, like CommonWell Health Alliance", and "e-Health exchange". Hospitals that responded "Often" or "Sometimes" to any of these options were considered sending and receiving data.

Integrating: We identified hospitals that integrated information into the EHR without manual intervention using the question "Does your EHR integrate the information contained in summary of care records received electronically (not eFax) without the need for manual entry?" Hospitals that responded "Yes, routinely" or "Yes, but not routinely" were considered as integrating data.

Correlation Matrix of Interoperability Domains

	Integrate	Send	Receive	Find/Query
Integrate	1.0000			
Send	0.2520	1.0000		
Receive	0.3753	0.5355	1.0000	
Find/Query	0.3550	0.2826	0.3761	1.0000

© 2022 Holmgren AJ et al. *JAMA Health Forum.*

eMethods 2. Full AHA Survey Questions for Alternative Payment Model Participation

We created dichotomous measures of participation in each of the three APMs. For Accountable Care Organizations, we used the question “Has your hospital or health care system established an accountable care organization (ACO)?” for 2014 – 2017, with hospitals that replied “Yes” considered to participate in an ACO for that year. For 2018, we used the question “Has your hospital or health care system established an accountable care organization (ACO)?” Hospitals that replied “My hospital currently leads an ACO” or “My hospital currently participates in an ACO (but is not its leader)” were considered to participate in an ACO that year.

For patient-centered medical homes, we used the question “Does your hospital have an established medical home program?” where hospitals that replied “Yes” were considered to participate in a PCMH. Finally, for bundled payments, we used the question “Does your hospital participate in a bundled payment program involving inpatient, physician, and/or post-acute care services where the hospital receives a single payment from a payer for a package of services and then distributes payments to participating care delivery organizations (such as a single fee for hospital and physician services for a specific procedure, e.g. hip replacement, CABG)?” where hospitals that responded “Yes” were considered to participate in a bundled payment program, for 2015 – 2018 (bundled payment data was not available for 2014). Hospitals that participated in one or more APMs in a year were considered an APM participant.

© 2022 Holmgren AJ et al. *JAMA Health Forum.*

eTable 1. Descriptive Statistics for Hospital Demographics and EHR Vendors

	2014		2015		2016		2017		2018	
	%	SD	%	SD	%	SD	%	SD	%	SD
**EHR Adoption**										
Less than Basic EHR	25.4%	0.44	16.4%	0.37	12.4%	0.33	5.8%	0.23	1.9%	0.14
Basic EHR	40.4%	0.49	44.3%	0.50	35.2%	0.48	39.3%	0.49	32.9%	0.47
Comprehensive EHR	34.2%	0.47	39.3%	0.49	52.4%	0.50	54.9%	0.50	65.2%	0.48
**RHIO Participation**										
Not a RHIO Member	41.3%	0.49	37.5%	0.48	33.5%	0.47	36.9%	0.48	28.0%	0.45
Participant in a RHIO	58.7%	0.49	62.5%	0.48	66.5%	0.47	63.1%	0.48	72.0%	0.45
**Hospital Size**										
Small Hospitals (<100 Beds)	50.7%	0.50	50.2%	0.50	50.2%	0.50	50.5%	0.50	50.0%	0.50
Medium Hospitals (100 - 399 Beds)	39.4%	0.49	39.7%	0.49	39.7%	0.49	39.4%	0.49	39.4%	0.49
Large Hospitals (>400 Beds)	9.9%	0.30	10.1%	0.30	10.0%	0.30	10.1%	0.30	10.6%	0.31
**Teaching Status**										
Non-Teaching Hospitals	71.5%	0.45	71.4%	0.45	69.8%	0.46	66.5%	0.47	64.4%	0.48
Teaching Hospitals	28.5%	0.45	28.6%	0.45	30.2%	0.46	33.5%	0.47	35.6%	0.48
**Health System Membership**										
Not a member of a health system	38.2%	0.49	36.4%	0.48	35.9%	0.48	34.8%	0.48	34.1%	0.47
Member of a health system	61.8%	0.49	63.6%	0.48	64.1%	0.48	65.2%	0.48	65.9%	0.47
**Location**										
Rural	41.8%	0.49	42.1%	0.49	42.1%	0.49	42.2%	0.49	40.8%	0.49
Urban	58.2%	0.49	57.9%	0.49	57.9%	0.49	57.8%	0.49	59.2%	0.49
Region: Northeast	12.4%	0.33	12.4%	0.33	12.5%	0.33	12.4%	0.33	12.4%	0.33
Region: West	19.8%	0.40	20.0%	0.40	20.0%	0.40	19.8%	0.40	19.7%	0.40
Region: Midwest	30.1%	0.46	30.3%	0.46	30.7%	0.46	30.5%	0.46	30.0%	0.46
Region: South	37.7%	0.48	37.4%	0.48	36.9%	0.48	37.2%	0.48	36.7%	0.48
**Alternative Payment Models**										
Accountable Care Organization	21.9%	0.41	26.9%	0.44	31.5%	0.46	35.9%	0.48	41.1%	0.49
Patient-Centered Medical Home	20.2%	0.40	21.8%	0.41	23.8%	0.43	16.0%	0.37	17.4%	0.38
Bundled Payments			12.6%	0.33	14.9%	0.36	14.2%	0.35	13.6%	0.34

	2014		2015		2016		2017		2018	
**EHR Vendor**	n	%	n	%	n	%	n	%	n	%
Allscripts / Eclipsys	86	3%	76	3%	74	3%	91	4%	139	5%
Cernere	410	15%	494	18%	567	21%	564	22%	582	22%
Epic	540	19%	604	22%	673	25%	752	29%	824	30%
GE	16	1%	7	0%	7	0%	7	0%	5	0%

© 2022 Holmgren AJ et al. *JAMA Health Forum.*

McKesson	248	9%	209	8%	194	7%	103	4%	41	2%
Meditech	677	24%	652	24%	612	23%	562	22%	592	22%
NextGen	29	1%	18	1%	8	0%	5	0%	3	0%
Harris Healthcare/QuadraMed	17	1%	22	1%	31	1%	20	1%	20	1%
Siemens	151	5%	70	3%	1	0%	0	0%	0	0%
Self-Developed	15	1%	10	0%	11	0%	2	0%	9	0%
Other	100	4%	65	2%	36	1%	36	1%	41	2%
Did Not Disclosee	7	0%	3	0%	3	0%	4	0%	5	0%
CPSI / Evident	261	9%	265	10%	138	5%	106	4%	227	8%
HMS	117	4%	101	4%	4	0%	0	0%	0	0%
Healthland	108	4%	111	4%	85	3%	79	3%	66	2%
Evideent	0	0%	0	0%	121	5%	111	4%	3	0%
Medhost	0	0%	0	0%	100	4%	89	3%	92	3%
Allscripts	0	0%	0	0%	0	0%	0	0%	1	0%
Prognosis	0	0%	0	0%	10	0%	5	0%	0	0%
Athenahealth	0	0%	0	0%	13	0%	29	1%	40	1%
MedWorx	0	0%	0	0%	2	0%	5	0%	3	0%
Azalea Health	0	0%	0	0%	0	0%	0	0%	10	0%

© 2022 Holmgren AJ et al. *JAMA Health Forum.*

eMethods 3. Technical Appendix and Robustness Tests for Two-Way Fixed Effects Design

Our primary specification is a two-way fixed effects model where the dependent variable is a binary indicator of whether or not a hospital reported engagement in all 4 domains of interoperability in a given year, and the independent variable of interest being whether a hospital participated in *any* alternative payment model in that year. We use hospital fixed effects to control for time-invariant unobserved confounding, and year fixed effects to control for the effect of the secular increase in interoperability over time. We also include a set of time-varying controls. Our primary analytic dataset is an unbalanced panel of hospitals from 2014 – 2018, which includes 3,914 unique hospitals and 13,864 hospital-year observations. All models include robust standard errors clustered at the hospital level.

There are two critical assumptions necessary for two-way fixed effects to produce an unbiased average treatment effect estimate. These are the *constant treatment effect* assumption and the *no unobserved time-varying confounders* assumption. In this technical appendix, we discuss in detail our choice to use two-way fixed effects and perform several robustness tests on our main specification. We then show empirical tests of these two assumptions, and employ a new estimator that relaxes the constant treatment effect assumption.

Interoperability by Always, Sometimes, and Never APM Participants

First, we wanted to compare our Exhibit 3 where we show APM vs non APM hospitals in repeated cross-sections over the years with a setup that compares hospitals who were *always* a member of an APM during our study period, those who were *sometimes* an APM member, and those who were *never* an APM participant.

© 2022 Holmgren AJ et al. *JAMA Health Forum.*

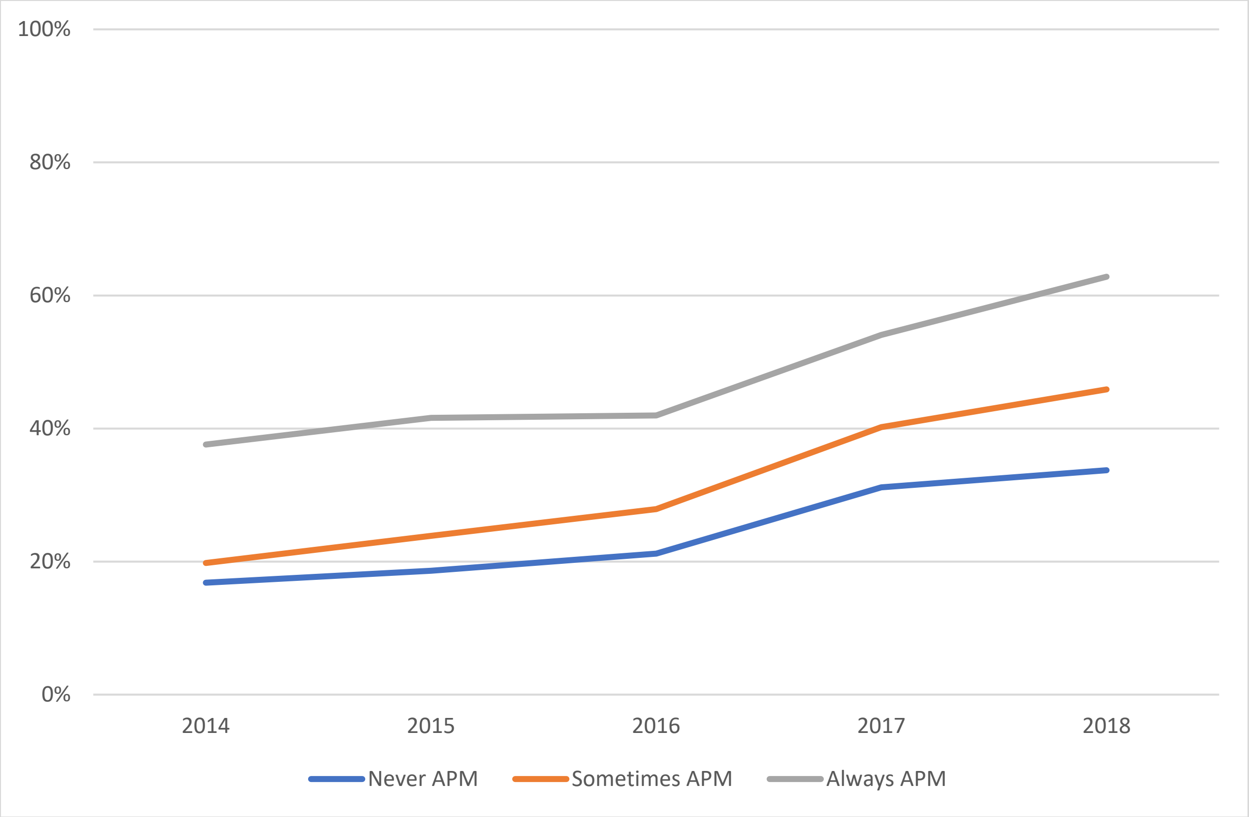

Variation in the Treatment Variable

First, we want to ensure that there are time-varying changes in the treatment variable, otherwise a fixed effects estimator will not have any variation to identify off of.

	Any APM	PCMH	Bundled Payments	ACO
2014	31.5%	20.2%	0.0%	21.9%
2015	39.0%	21.8%	12.6%	26.9%
2016	43.6%	23.8%	14.9%	31.5%
2017	50.7%	16.0%	14.2%	35.9%
2018	44.8%	17.4%	13.6%	41.1%

There is significant churn in and out of each alternative payment model, as well as in our binary measure of hospital participation in any APM that year.

Hausman Test: Do We Need Fixed Effects?

Our first diagnostic test is to determine whether we need to use hospital-level fixed effects, or if a random effects will produce an unbiased estimate. To do this, we use a Hausman test to

© 2022 Holmgren AJ et al. *JAMA Health Forum.*

determine whether our *consistent* estimator (fixed effects) produces differences in coefficients that are systematically different than our *efficient* (random effects) estimator.

```
                 ---- Coefficients ----
             |      (b)          (B)            (b-B)     sqrt(diag(V_b-V_B))
             |     fixed        random       Difference          S.E.
-------------+----------------------------------------------------------------
basicNotComp |    .0119557     .0878548       -.0758991         .0050987
     compEHR |    .0831265     .2687162       -.1855897         .0076305
      RHIO_1 |    .1220969     .1638214       -.0417246         .0058554
   sysMember |    .0023925     .1360596       -.1336671         .0228486
      AnyAPM |    .0179627     .0556758       -.0377131         .0063443
------------------------------------------------------------------------------
                          b = consistent under Ho and Ha; obtained from reghdfe
           B = inconsistent under Ha, efficient under Ho; obtained from xtreg

    Test:  Ho:  difference in coefficients not systematic

                  chi2(5) = (b-B)'[(V_b-V_B)^(-1)](b-B)
                          =      687.26
                Prob>chi2 =      0.0000
```

The results of the Hausman test reject the null hypothesis of no systematic differences in coefficients, indicating that a random effects model would be biased.

Main Specification: Two-Way Fixed Effects with Time-Varying Covariates Full Model – One Treatment

DV: All 4 Interop Domains	Coef.	p-value	95% CI
**Alternative Payment Model Participation**	**0.01**	**0.30**	**-0.01 – 0.03**
Basic EHR	0.01	0.30	-0.01 – 0.03
Comprehensive EHR	0.08	<0.001	0.06 – 0.11
RHIO Participation	0.12	<0.001	0.10 – 0.15
Member of a Health System	<0.01	0.92	-0.05 – 0.05

We use Stata's `reghdfe` command to iteratively remove singleton groups and ensure they do not bias standard error calculations. In this specification we find a null effect with a tight 95% confidence interval on the dummy variable for APM participation.

Alternative Specification: Two-Way Fixed Effects with Time-Varying Covariates Full Model – Individual APMs

DV: All 4 Interop Domains	Coef.	p-value	[95% Conf.

© 2022 Holmgren AJ et al. *JAMA Health Forum.*

Patient Centered Medical Home	0.01	0.69	-0.02 – 0.03
Bundled Payment Program	-0.01	0.45	-0.04 – 0.02
Accountable Care Organization	0.01	0.30	-0.01 – 0.04
Basic EHR	0.01	0.30	-0.01 – 0.03
Comprehensive EHR	0.08	<0.001	0.06 – 0.11
RHIO Participation	0.12	<0.001	0.10 – 0.15
Member of a Health System	<0.01	0.94	-0.05 – 0.05

Using the same setup as our previous model, but rather than a binary dummy variable for participation in any of the 3 APMs, we disaggregate them into the 3 individual APMs. We once again find a null effect for each, with small confidence intervals.

Alternative Specification: Two-Way Fixed Effects with Time-Varying Covariates Full Model – All Permutations

DV: All 4 Interop Domains	Coef.	p-value	[95% Conf.	Interval]
Basic EHR	0.01	0.30	-0.01	0.03
Comprehensive EHR	0.08	0.00	0.06	0.11
RHIO Participation	0.12	0.00	0.10	0.15
System Membership	0.00	0.97	-0.05	0.05
PCMH	-0.02	0.32	-0.06	0.02
Bundled Pay	-0.02	0.45	-0.07	0.03
PCMH + Bundled Pay	0.01	0.85	-0.07	0.09
ACO	0.01	0.38	-0.02	0.04
PCMH + ACO	0.04	0.09	-0.01	0.09
Bundled Pay + ACO	-0.01	0.78	-0.08	0.06
All 3	-0.01	0.84	-0.11	0.09

In this setup, we interact the indicators for hospital participation in all 3 APMs to fully saturate the model and show all possible hospital APM participation. Once again, we find a null effect.

Alternative Setup: Two-Way Fixed Effects with Time-Varying Covariates Full Model – Balanced Panel

In this setup, we completely balance our data panel and keep only hospitals who responded to the survey in all 5 years. In this setup we have 1,462 hospitals responding in every year.

© 2022 Holmgren AJ et al. *JAMA Health Forum.*

DV: All 4 Interop Domains	Coef.	p-value	[95% Conf.	Interval]
Basic EHR	0.01	0.46	-0.02	0.05
Comprehensive EHR	0.06	0.00	0.02	0.10
RHIO Participation	0.15	0.00	0.11	0.18
System Membership	0.00	0.90	-0.07	0.06
**APM Participation**	**0.02**	**0.21**	**-0.01**	**0.05**

Once again, we find a precisely estimated null effect on the effect of APM participation on interoperability engagement.

We find similar results to our unbalanced panel in the other 2 models as well:

DV: All 4 Interop Domains	Coef.	p-value	[95% Conf.	Interval]
Basic EHR	0.01	0.44	-0.02	0.05
Comprehensive EHR	0.06	0.00	0.02	0.10
RHIO Participation	0.15	0.00	0.11	0.18
System Membership	0.00	0.89	-0.07	0.06
**PCMH**	**0.00**	**0.85**	**-0.04**	**0.03**
**Bundled Payment**	**-0.01**	**0.68**	**-0.04**	**0.03**
**ACO**	**0.02**	**0.26**	**-0.01**	**0.05**

DV: All 4 Interop Domains	Coef.	p-value	[95% Conf.	Interval]
Basic EHR	0.01	0.44	-0.02	0.05
Comprehensive EHR	0.06	0.00	0.02	0.10
RHIO Participation	0.15	0.00	0.11	0.18
System Membership	0.00	0.88	-0.07	0.06
PCMH	0.00	0.96	-0.05	0.05
Bundled Pay	0.00	0.94	-0.07	0.06
PCMH + Bundles	-0.02	0.69	-0.12	0.08
ACO	0.02	0.40	-0.02	0.05
PCMH + ACO	0.01	0.83	-0.06	0.07
Bundled Pay + ACO	0.01	0.83	-0.07	0.09
All 3	-0.01	0.87	-0.14	0.11

© 2022 Holmgren AJ et al. *JAMA Health Forum.*

Alternative Specification: Expressing the Dependent Variable as a Count of Interoperability Capabilities

In this model, rather than expressing the dependent variable as a binary indicator of whether a hospital is engaged in all four domains of interoperability, we model the outcome variable as a count variable of hospital engagement in 0 – 4 interoperability domains. We once again use a linear model with two-way fixed effects – the same setup as our main specification – to estimate the impact of APM participation on the marginal increase in interoperability domain engagement.

DV: Count of Interop Domains	Coef.	p-value	[95% Conf.	Interval]
Basic EHR	0.18	<0.001	0.10	0.26
Comprehensive EHR	0.40	<0.001	0.31	0.49
RHIO Participation	0.58	<0.001	0.51	0.65
System Membership	0.06	0.41	-0.08	0.21
**APM Participation**	**0.04**	**0.19**	**-0.02**	**0.10**

We find similar results to our primary specification, suggesting no statistically significant effect of APM participation on the number of interoperability domains a hospital is engaged in.

Alternative Specification: Expressing the Dependent Variable as 3 Domains (Without Integration)

In this model, rather than using a dichotomous measure of all 4 domains of interoperability, we create a binary measure of hospital engagement in 3 domains – finding, sending, and receiving data, as integration of data as a conceptually different capability despite being an important aspect of data exchange. We once again use a linear model with two-way fixed effects to estimate the impact of APM participation on the marginal increase in interoperability engagement.

DV: 3 Interop Domains	Coef.	p-value	[95% Conf.	Interval]
Basic EHR	0.03	0.02	0.01	0.06
Comprehensive EHR	0.12	<0.001	0.09	0.16
RHIO Participation	0.15	<0.001	0.12	0.17
System Membership	0.03	0.31	-0.03	0.09
**APM Participation**	**-0.01**	**0.38**	**-0.03**	**0.01**

We find qualitatively similar results, suggesting that measuring interoperability without the integration component does not change the association with alternative payment model participation.

© 2022 Holmgren AJ et al. *JAMA Health Forum.*

Alternative Specification: Changing Comparison Groups

While the fixed effects design is often called a *within* estimator, we wanted to ensure our comparison group was accurate. We ran robustness tests on our model where we dropped all hospitals who were *always treated* – that is, they were within an APM the entire sample period, and can be considered "left censored" with respect to the treatment variable.

DV: All 4 Interop Domains	Coef.	p-value	[95% Conf.	Interval]
Basic EHR	0.00	0.98	-0.02	0.02
Comprehensive EHR	0.04	0.01	0.01	0.07
RHIO Participation	0.11	0.00	0.09	0.14
System Membership	0.00	0.87	-0.05	0.06
**APM Participation**	**0.02**	**0.13**	**-0.01**	**0.04**

We found similar results as our main specification.

Alternative Specification: Dose-Response Effect

We wanted to evaluate whether there was a dose-response effect, that is, are hospitals participating in *more* APMs more likely to become interoperable upon joining a marginal additional APM?

DV: All 4 Interop Domains	Coef.	p-value	[95% Conf.	Interval]
Count of APMs (0 - 3)	0.00	0.66	-0.01	0.02
Basic EHR	0.01	0.29	-0.01	0.03
Comprehensive EHR	0.08	0.00	0.06	0.11
RHIO Participation	0.12	0.00	0.10	0.15
System Membership	0.00	0.91	-0.05	0.05

This is also true specifying the model more flexibly:

DV: All 4 Interop Domains	Coef.	p-value	[95% Conf.	Interval]
Number of APMs				
0	Ref			
1	-0.0006	0.96	-.02	0.02
2	0.01	0.51	-0.02	0.05

© 2022 Holmgren AJ et al. *JAMA Health Forum.*

3	0.003	0.90	-0.05	0.06
Basic EHR	0.01	0.29	-0.01	0.03
Comprehensive EHR	0.08	0.00	0.06	0.11
RHIO Participation	0.12	0.00	0.10	0.15
System Membership	0.00	0.91	-0.05	0.05

Once again, we find a null effect with small confidence intervals on the effect of joining a marginal APM.

Alternative Specification: Removing APM "Leavers"

There is churn in and out of alternative payment models over the course of the sample period. To ensure our results our robust, we wanted to estimate the effect of joining an APM on interoperability *without* the subset of hospitals that would then leave the APM. In this analysis, we discarded any hospitals that were participating in any APM and then left the APM in at any point in the study period.

DV: All 4 Interop Domains	Coef.	p-value	[95% Conf.	Interval]
APM Participation	0.04	0.14	-0.01	0.08
Basic EHR	0.01	0.71	-0.03	0.05
Comprehensive EHR	0.06	0.01	0.01	0.11
RHIO Participation	0.14	0.00	0.10	0.19
System Membership	0.01	0.70	-0.05	0.08

Alternative Specification: Removing Interoperability "De-Adopters"

While the majority of hospitals, once they begin to participate in interoperability, stay that way for the remainder of our study period, due to either different interpretations of the response questions that make up our interoperability measures or actual reduction or de-adoption of data exchange, it may be possible some hospitals do not stay interoperable. In this specification we have excluded any hospital that reported that they engaged in all 4 domains of interoperability and then reported they did not in a following year.

DV: All 4 Interop Domains	Coefficient	p-value	[95% conf.	interval]
**APM Participation**	**0.01**	**0.41**	**-0.01**	**0.03**
Basic EHR	-0.01	0.46	-0.03	0.01
Comprehensive EHR	0.05	0.00	0.03	0.08
RHIO Participation	0.08	0.00	0.06	0.10
System Membership	-0.04	0.15	-0.09	0.01

© 2022 Holmgren AJ et al. *JAMA Health Forum.*

Diagnostic Test: Heterogenous Treatment Effects

Recent empirical research on the use of two-way fixed effects (hereafter TWFE) has highlighted the possible shortcoming involved if there are heterogenous treatment effects. This is because these estimators identify weighted sums of average treatment effects (ATE) in each group with weights that may be negative, and those negative weights may create an instance where the estimand is negative despite all ATEs being positive. We use the diagnostic tests outlined in de Chaisemartin and D'Haultfœuille (2020) to determine whether our model is susceptible to this bias. If the proportion of negative weights is high, a weighted fixed effects estimator is necessary.

```
. twowayfeweights All4 ID year AnyAPM, type(feS) controls(year ID)
Under the common trends, treatment monotonicity, and stable treatment effect assumptions,
beta estimates a weighted sum of 7547 LATEs.
7543 LATEs receive a positive weight, and 4 receive a negative weight.
The sum of the negative weights is equal to -.00262477.
beta is compatible with a DGP where the average of those LATEs is equal to 0,
while their standard deviation is equal to .01579132.
beta is compatible with a DGP where those LATEs all are of a different sign than beta,
while their standard deviation is equal to .32574448.

.
end of do-file

. do "/var/folders/n7/f_99s4g56dv8_ppcq5fhvs0wmxs957/T//SD01581.000000"

. twowayfeweights All4 ID year AnyAPM, type(feTR)
Under the common trends assumption, beta estimates a weighted sum of 12945 ATTs.
12280 ATTs receive a positive weight, and 665 receive a negative weight.
The sum of the negative weights is equal to -377.13425.
beta is compatible with a DGP where the average of those ATTs is equal to 0,
while their standard deviation is equal to .00626299.
beta is compatible with a DGP where those ATTs all are of a different sign than beta,
while their standard deviation is equal to ..
```

We find that under two different sets of assumptions, only a very small proportion of the weighted sums are negative. The results of this diagnostic test indicate we do not need to be concerned that our estimate is biased by heterogenous treatment effects.

Event Study Framework

In this framework, we standardize the year a hospital joins an APM at t=0 and plot the coefficient estimates and 95% confidence intervals from t = -4 to t = 4, leaving out t = -1 as a comparison group. All event study regression models include two-way fixed effects and our time-varying covariates. This visual display also allows us to see whether APM participation appears to incentivize interoperability several years post-joining the APM. We use the method described in Freyaldenhoven S, Hansen C, Pérez JP, Shapiro JM. Visualization, Identification, and Estimation in the Linear Panel Event-Study Design. National Bureau of Economic Research; 2021. doi:10.3386/w29170, using the `xtevent` Stata package to estimate the linear models,

robust standard errors clustered at the hospital level, and plot the point estimates and 95% confidence intervals.

Independent Variable: Any APM

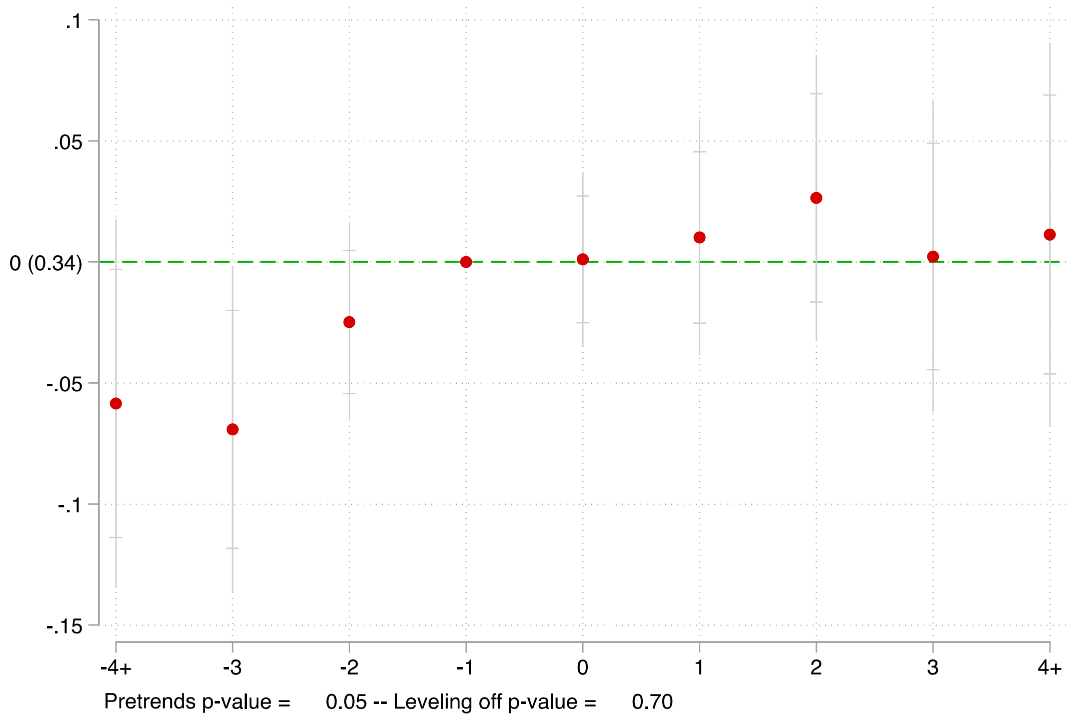

ACO

© 2022 Holmgren AJ et al. *JAMA Health Forum.*

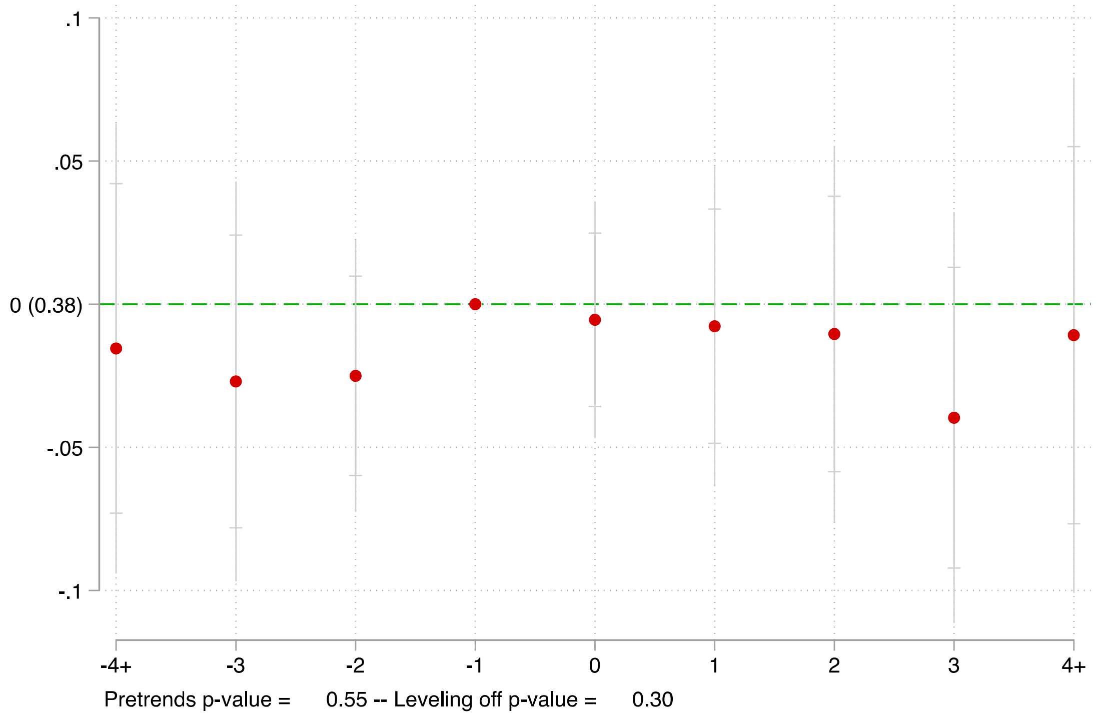

Bundled Payments

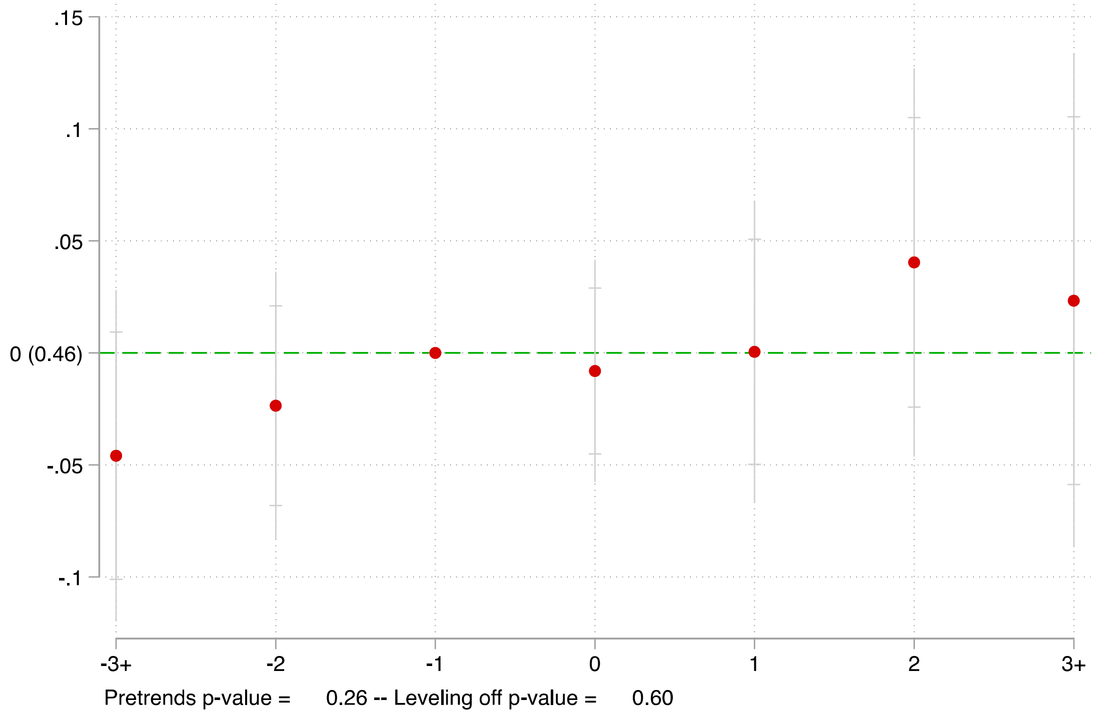

Patient-Centered Medical Home (PCMH)

© 2022 Holmgren AJ et al. *JAMA Health Forum.*

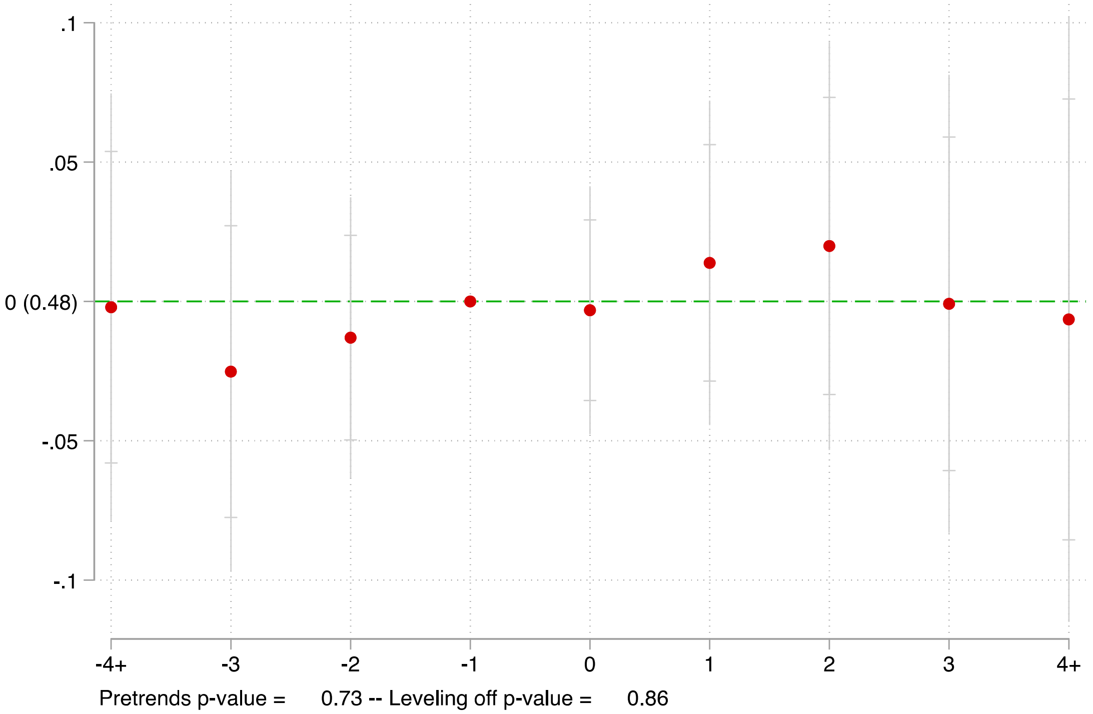

In this framework we once again find no evidence that APM participation has an effect on interoperability engagement.

Callaway and Sant'Anna

Next, we take advantage of recent econometric advances in estimating treatment effects in difference-in-differences setups with varying treatment times by employing a new estimator by Callaway and Sant'Anna (2020). This analysis estimates a group-time average treatment effect on the treated, for groups of hospitals that first reported APM participation in 2015, 2016, 2017, and 2018. This estimator from Callaway & Sant'Anna relaxes the traditional unconditional parallel pre-trends assumption to an assumption of parallel pre-trends across treated units and never-treated units conditional on covariates, justified by the observed differences in the characteristics of hospitals that never participate in APMs compared to those that do. This estimator applies weighting for hospitals that join APMs at different times, measures dynamic treatment effects similar to an event study regression framework, and adjust pre-intervention estimates for selective treatment timing. Below is a graph showing the event study plot from the Callaway and Sant'Anna estimator where the treatment is joining any APM. No years appear to be left out as the comparison group as in this estimator the dynamic treatment effects are always to the t-1 period (the year prior to joining an APM), except for pre-treatment years which are in comparison to the year immediately before. In this estimator we use a balanced panel of hospitals.

© 2022 Holmgren AJ et al. *JAMA Health Forum.*

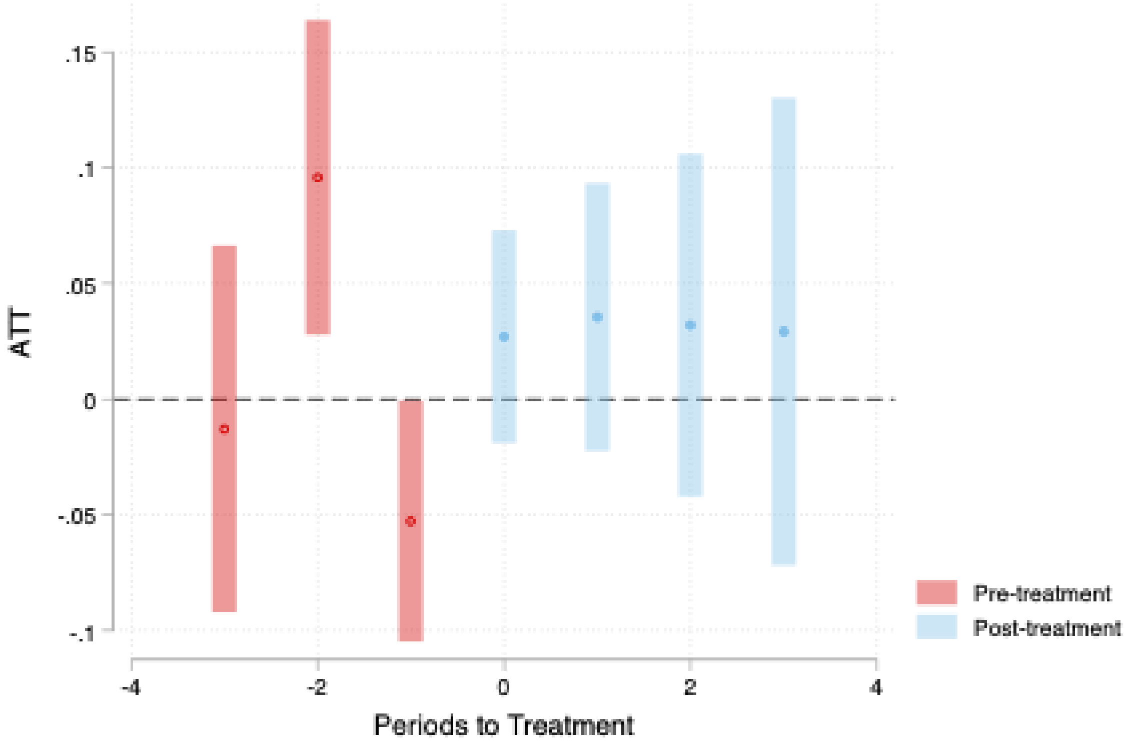

In this estimator, we once again find a similar result – no statistically significant impact of joining an APM on interoperability. Instead, consistent with a potential explanation of how to reconcile our findings with previous cross-sectional evidence that found an association between APM participation and interoperability, we find that APM hospitals were more likely to engage in interoperability prior to joining the APM. It may be that an unobserved confounder is associated with early adoption of both data sharing and voluntary alternative payment models.

Testing the Identification Assumptions

Relaxing the Constant Treatment Effect Assumption

To further test the robustness of our models, we use a new estimator, the *two-way fixed-effects counterfactual estimator* (hereafter FEct) developed by Liu, Wang, & Xu (2020). FEct is similar to the traditional two-way fixed effects estimator used in `reghdfe`, but relaxes the constant treatment assumption. This model requires a balanced panel and discards data with no time under the control (that is, all hospitals who were always treated – in an alternative payment model – are dropped.)

We estimate this model and plot coefficient estimates and 95% confidence intervals (from bootstrapped standard errors) of the average treatment effect below at each year prior to and after joining an APM and find a similar null result:

© 2022 Holmgren AJ et al. *JAMA Health Forum.*

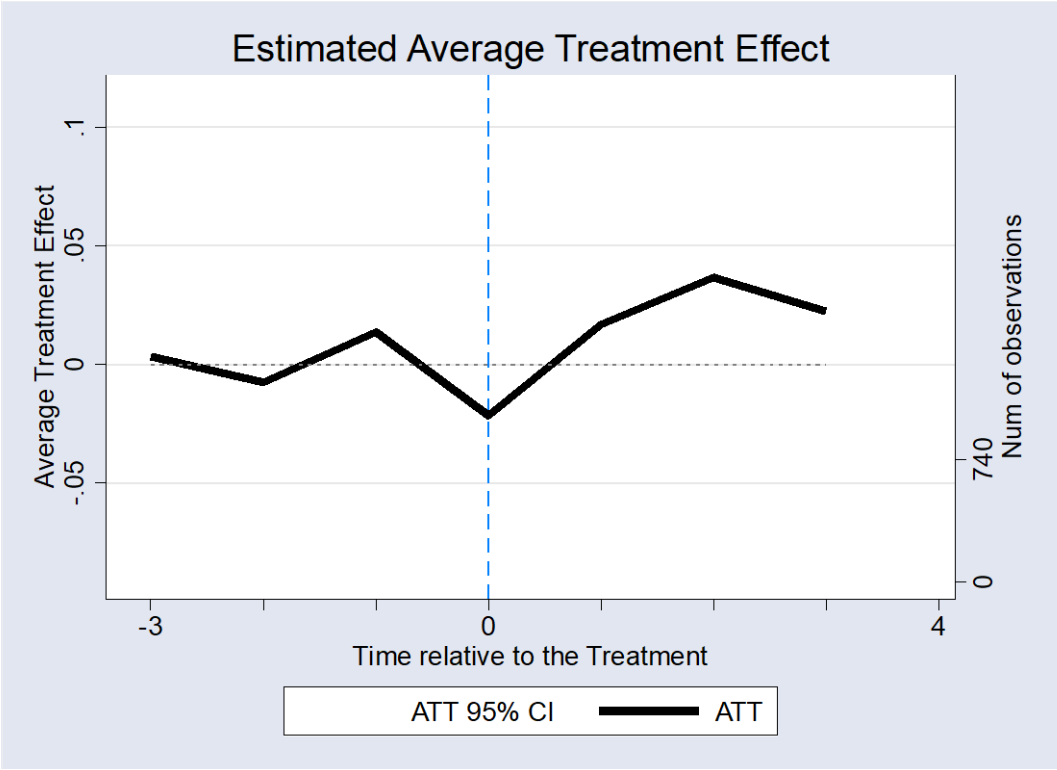

Additional Diagnostic Tests

The No Time Varying Confounders Test

The FEct model setup also allows two convenient tests of our identifying assumption of *no time-varying confounders*. The first is a variant on the Wald (*F* statistic) test – a joint test on a set of null hypotheses that the average of residuals for any pre-treatment period is zero. A statistically significant result on this test would indicate that there is evidence that we should reject the null hypothesis of an ATE = 0 in pre-treatment periods and suggest that time-varying confounders exist.

We conduct this Wald test in a similar setup to our FEct model above and find a p-value of 0.4, which can be interpreted that there is no evidence of time-varying confounders in our data.

© 2022 Holmgren AJ et al. *JAMA Health Forum.*

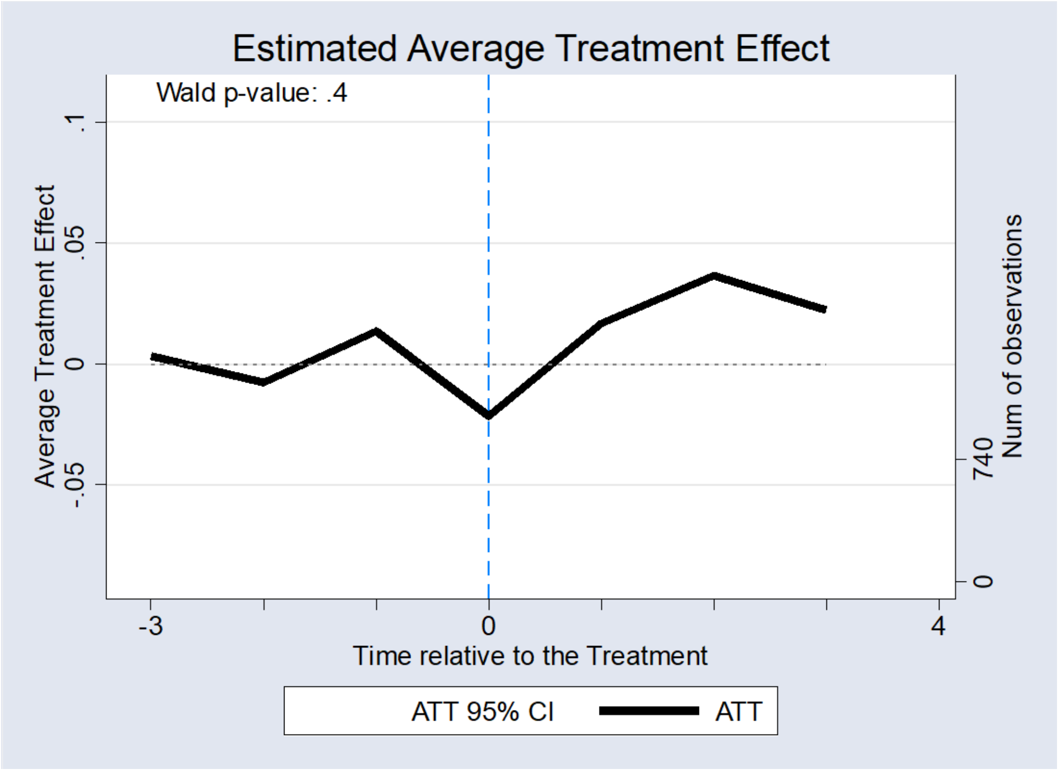

A second and related test is a variant of the *equivalence test* first proposed in Hartman and Hidalgo (2018). In this test we reverse the null hypothesis of the Wald test, and test if we find any evidence that pre-treatment residuals are non-zero. We use the default equivalence bound calculation from Hartman and Hidalgo of 0.36 * the standard deviation of the residualized non-treated outcome. We then check whether the minimum bounds of our pre-treatment period estimates are within the range of the equivalence bounds.

© 2022 Holmgren AJ et al. *JAMA Health Forum.*

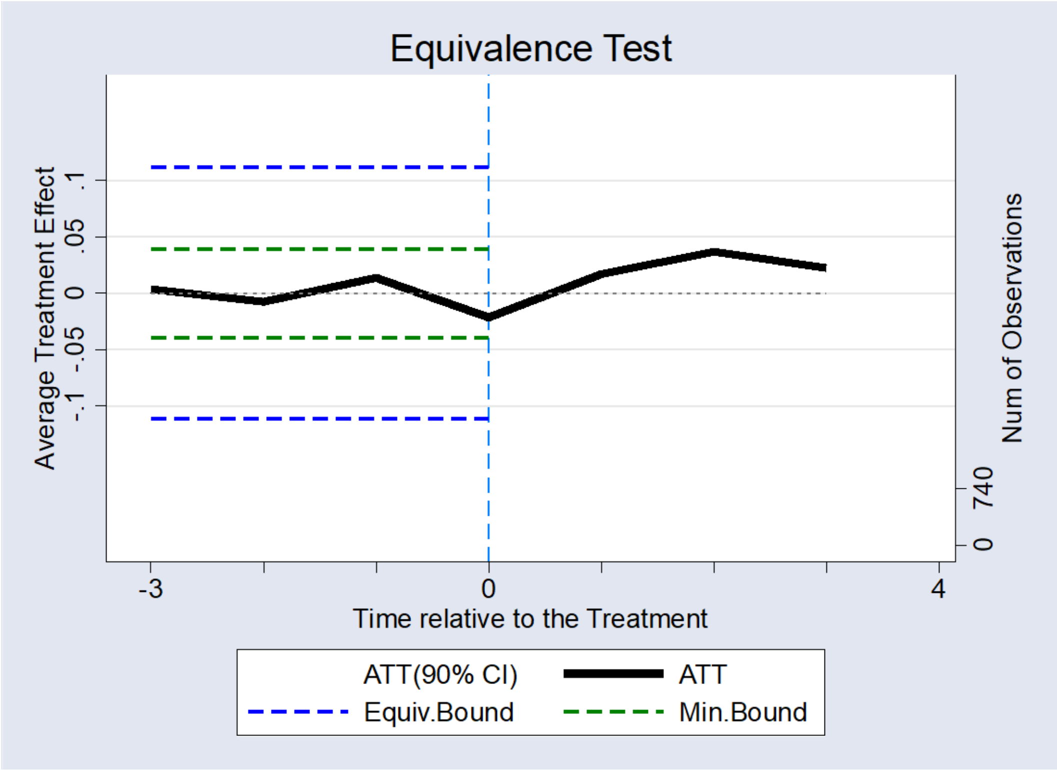

We find our data passes this test using the FEct model, suggesting that equivalence holds and that we find no evidence of time-varying confounders. Further, we are well within the equivalence bounds, suggesting that even on a very conservative estimate, we find no evidence of time-varying confounders.

© 2022 Holmgren AJ et al. *JAMA Health Forum.*

eTable 2. Association of APM Participation and Number of Hospital-Reported Barriers to Interoperability

DV: Sum of Reported Barriers to Interoperability	Coef.	p-value	[95% Conf.	Interval]
Did Not Participate in an APM in 2018	Ref			
**Participated in an APM in 2018**	**0.51**	**<0.001**	**0.33**	**0.69**
Not Engaged in All Four Domains of Interoperability	Ref			
Engaged in All Four Domains of Interoperability	-0.37	<0.001	-0.55	-0.19
Less than Basic EHR	Ref			
Basic EHR	0.32	0.40	-0.43	1.06
Comprehensive EHR	0.13	0.72	-0.61	0.88
Small Hospitals Fewer than 100 Beds	Ref			
Medium Hospitals Between 100 - 399 Beds	0.09	0.45	-0.14	0.32
Large Hospitals Over 400 Beds	0.26	0.13	-0.08	0.60
Non-Teaching Hospitals				
Teaching Hospitals	0.10	0.37	-0.12	0.33
Non-System Hospitals				
Health System Member Hospitals	-0.23	0.03	-0.43	-0.02
Hospitals Located in Rural Areas	Ref			
Hospitals Located in Urban Areas	0.06	0.58	-0.16	0.28
Located in the Northeastern US	Ref			
Located in the Western US	0.41	0.01	0.09	0.73
Located in the Midwestern US	-0.10	0.47	-0.36	0.16
Located in the Southern US	-0.20	0.13	-0.45	0.06

Notes: Results from an ordinary least squares model on 2018 data only, using robust standard errors. Dependent variable is the sum of hospital reported barriers in 2018.

eTable 3. Hospital Reported Barriers to Interoperability on Full Hospital Sample (2018)

Barrier	All Hospitals	Non-APM	APM	p-value
Experience greater challenges exchanging (e.g. sending/receiving data) across different vendor platforms	78.2%	77.0%	79.3%	0.21
There are providers whom we share patients with that don't typically exchange patient data with us	66.2%	67.1%	65.4%	0.38
**Providers we would like to electronically send patient health information to have an EHR; however, it lacks the technical capability to receive the information**	**55.5%**	**52.9%**	**58.2%**	**0.01**
**Difficult to locate the address of the provider to send the information (e.g. lack of provider directory)**	**55.1%**	**49.6%**	**60.7%**	**<0.001**
**Difficult to match or identify the correct patient between systems**	**49.3%**	**42.0%**	**56.7%**	**<0.001**
**Providers we would like to electronically send patient health information to do not have an EHR or other electronic system with capability to receive the information**	**47.2%**	**44.3%**	**50.2%**	**0.01**
We have to pay additional costs to send/receive data with care settings/organizations outside our system	42.4%	42.5%	42.4%	0.97
**Many recipients of our electronic care summaries (e.g. CCDA) report that the information is not useful**	**42.3%**	**37.2%**	**47.5%**	**<0.001**
**We had to develop customized interfaces in order to electronically exchange health information**	**37.7%**	**40.9%**	**34.8%**	**0.01**
Cumbersome workflow to send (not eFax) the information from our EHR system	26.3%	27.3%	25.4%	0.27
**No technical capability to electronically receive from outside providers**	**19.2%**	**23.1%**	**15.2%**	**<0.001**
**No technical capability to electronically send to outside providers**	**9.2%**	**12.2%**	**6.0%**	**<0.001**

© 2022 Holmgren AJ et al. *JAMA Health Forum.*

eTable 4. Hospital Reported Barriers to Interoperability, Limited to Hospitals Who Engaged in the Send and Receive Domain (2018)

Barrier	All Hospitals	Non-APM	APM	p-value
Experience greater challenges exchanging (e.g. sending/receiving data) across different vendor platforms	78.3%	77.3%	79.0%	0.42
There are providers whom we share patients with that don't typically exchange patient data with us	69.6%	72.9%	66.7%	0.004
Providers we would like to electronically send patient health information to have an EHR; however, it lacks the technical capability to receive the information	59.4%	58.4%	60.3%	0.40
Difficult to locate the address of the provider to send the information (e.g. lack of provider directory)	56.8%	50.6%	62.0%	<0.001
Difficult to match or identify the correct patient between systems	51.8%	44.7%	57.8%	<0.001
Providers we would like to electronically send patient health information to do not have an EHR or other electronic system with capability to receive the information	48.8%	45.8%	51.4%	0.01
Many recipients of our electronic care summaries (e.g. CCDA) report that the information is not useful	44.7%	40.0%	48.7%	<0.001
We have to pay additional costs to send/receive data with care settings/organizations outside our system	42.5%	43.1%	42.0%	0.65
We had to develop customized interfaces in order to electronically exchange health information	37.4%	41.0%	34.9%	0.01
Cumbersome workflow to send (not eFax) the information from our EHR system	23.7%	24.8%	22.7%	0.28
No technical capability to electronically receive from outside providers	12.3%	14.0%	10.9%	0.05
No technical capability to electronically send to outside providers	5.0%	6.4%	3.8%	0.007

© 2022 Holmgren AJ et al. *JAMA Health Forum.*
